# Supplementary material for: Anomalous magnetoresistance by breaking ice rule in Bi2Ir2O7/Dy2Ti2O7 heterostructure
Source: Nat Commun. 2023 Mar 14;14:1404. doi: 10.1038/s41467-023-36886-2 (PMC10014844; doi:10.1038/s41467-023-36886-2)
Supplement: Supplementary file 1 — Supplementary Information [file 41467_2023_36886_MOESM1_ESM.pdf]

Supplementary Information for

Anomalous Magnetoresistance by Breaking Ice Rule in $Bi_2Ir_2O_7/Dy_2Ti_2O_7$ Heterostructure

H. Zhang[1†], C. K. Xing[1†], K. Noordhoek[1†], Z. Liu[2], T. H. Zhao[3], L. Horák[4], Q. Huang[1]. L. Hao[1], J. Yang[1], S. Pandey[1], E. Dagotto[1,5], Z. Jiang[3], J. -H. Chu[2], Y. Xin[6], E. S. Choi[6], H. D. Zhou[1*] and J. Liu[1*]

[1]Department of Physics and Astronomy, University of Tennessee; Knoxville, TN, USA, 37996
[2]Department of Physics, University of Washington, Seattle; WA, USA, 98195
[3]School of Physics, Georgia Institute of Technology, Atlanta; GA, USA, 30332
[4]Charles University; Prague, Czechia,11636
[5]Materials Science and Technology Division, Oak Ridge National Laboratory; Oak Ridge, TN, USA, 37831
[6]National High Magnetic Field Laboratory, Florida State University; Tallahassee, FL, USA, 32310

†equal contribution; *corresponding author
Email: jianliu@utk.edu
hzhou10@utk.edu

Supplementary Note 1: Sample Information

The bulk lattice constants of the compounds involved in this study are displayed in Supplementary Table 1 below. Information about sample BD1 to BD6 can be found in Supplementary Table 2. Sample BD1 to BD6 are BIO/DTO heterostructures with the BIO thickness of 3.4nm, 3.2nm, 3.9nm, 4.0nm, 23.8nm and 41.4nm, respectively. The complete angle-dependent magnetoresistance (MR) measurement shown in the main text was performed on sample BD1. The MR data of samples with similar BIO thickness at 0.03K with **B**//[111] (Supplementary Fig.2f) demonstrates excellent reproducibility of the MR anomaly. The height, the relative height, the width, and the field position of the MR anomaly of these samples are extracted and listed in Table S2. The small quantitative differences are likely due to a combination of the slightly different dimensions of the DTO substrates, the intrinsic width of the transition in different DTO crystals, and the high sensitivity of the transition to misalignment of the field direction (see more discussions below). The size of the anomaly is decreased and eventually vanished when significantly increasing the BIO thickness (Supplementary Fig.3a).

Supplementary Note 2: X-ray reflectivity measurements

The film thickness is extracted from fitting the X-ray reflectivity curve (XRR). Figure S2b&c show a representative result fitted with a model of one homogeneous layer on a substrate. The calculation of the theoretical curve was based on the dynamical theory of X-ray scattering applied on the model of a perfectly flat homogeneous slab of given thickness grown on a perfectly flat substrate. The surface and interface roughness were included in the model using the distorted-wave Born approximation approach. The model parameters (explicitly the layer density, the substrate density, the layer thickness, and the root mean square roughness of the surface and of the interface) were numerically refined to fit the experimental XRR curve.

Supplementary Note 3: Supplementary transport measurements

It has been shown that the BIO metallicity will degrade when grown as ultrathin films likely due to weak localization and/or disorder-enhanced electron-electron interaction[32]. Indeed, the temperature-dependent resistivity of sample BIO/DTO (BD1) (i.e., the 3.4nm sample for the MR data in the main text) in Supplementary Fig.2d shows a weakly increasing resistivity when lowering the temperature. In addition, the MR of the same sample from 30 K down to 2K was

measured and the results are displayed in Supplementary Fig.2e. At higher temperatures, an overall positive MR is observed which is consistent with the literature[32]. As the temperature decreases, a negative component eventually starts to emerge around zero field and becomes stronger and stronger. The field range of the negative component also expands at the same time and eventually covers the entire measured field range at 0.03K, which is why the background MR in Figure 2 appears to be negative. Similar negative MR at ultralow temperatures was observed in other samples as well, including the BIO/ $Y_2Ti_2O_7$ (BY1) reference sample discussed below. While the exact nature of this negative region is not yet clear, it may be related to the electron-electron interaction correction at 2D since it is more significant in the thinner films regardless of the substrate. Nevertheless, we do not find any strong correlation between the MR anomaly and the temperature/field range the negative region covers.

The MR result at 0.03K shown in the Figure 2d of the main text are plotted again in Supplementary Fig.2g but in the measured resistivity of the BIO film instead of the MR ratio and without the vertical shift.

Supplementary Note 4: Shift of the critical field B_c when the field direction deviates [111] axis

The pyrochlore lattice geometry and frustrated local Ising axes give rise to multiple non-orthonormal principal axes in this system. As a result, the critical field B_c from Kagome ice-to-saturated ice state generally does not follow any simple cosine relation when its direction deviates the <111> axis[33,34]. In the present case where the field is rotating in the [111]-[1-10] plane, B_c can be deduced as $B_c = \frac{0.6J_{eff}/\mu_B}{cos|\theta|-\sqrt{6}sin|\theta|} + B_{dimagnetization}$, where $J_{eff} = 1.01K$ represents the effective exchange interaction in the nearest-neighbor spin-ice model[33]. θ is the field angle away from the [111] axis. One can see that B_c diverges around 20°, which is a signature of frustration of this system. We show the MR anomaly position from Figure 2b and the calculated B_c as functions of θ in Figure S5a below with $B_{dimagnetization} = 0.7$ T. One can see that the anomaly position and the calculated B_c have overall consistent angle dependence except a horizontal shift of ~2°. This shift is due to the mechanical misalignment of the sample rotator at station SCM1 at NHMFL. Downshifting the experimental angle by 2° resolves this issue as shown in Supplementary Fig.5b.

Supplementary Note 5: Magnetoresistance of BIO/ $Y_2Ti_2O_7$ heterostructure

To further confirm the MR anomaly in BIO/DTO is associated with the ice-rule-breaking in DTO. We have also prepared a reference sample where a 3.7nm BIO film was deposited on a (111)-oriented $Y_2Ti_2O_7$ (YTO) single crystal substrate (Supplementary Fig.6). The measured MR of such BIO/YTO (BY1) heterostructure at 0.03K is shown in Supplementary Fig.6f, where it is indeed isotropic between **B**//[111] and **B**//[1-10] without any extra feature, consistent with the fact that Dy is replaced with the nonmagnetic Y ion. One can see that the transport behavior is similar between the BIO/DTO and BIO/YTO heterostructures, except the MR anomaly near the ice-rule-breaking transition. It suggests their difference in the epitaxial strain is not expected to play any significant role in accounting for the physics of the anomaly. Besides, this similarity indicates that there is no fundamental change to the properties of the BIO film or any significant proximity-induced spin-ice structure in BIO when grown on DTO. This is reasonable because the spin ice structures rely on dipolar interaction of large and strong Ising spins, both of which are absent in BIO. Instead, the influence of DTO occurs when the BIO carriers reach the interface and are scattered by the fluctuating Dy moments during the transition as discussed in the main text. Such mechanism does not require a spin ice structure inside BIO and is yet consistent with our observations.

Supplementary Note 6: Ultralow temperature measurement procedure

The measurements which involve changing the magnetic field at the National High Magnetic Field Laboratory were performed with a very slow field sweeping rate at 0.2T/minute and a significant wait time of more than 60 seconds at each field before taking the data, with the field stepsize to be 0.05T. This necessary procedure allows the sample to reach thermal equilibrium, and also helps mitigate the uncontrollable flux jump in the magnet during continuous field sweep. But it precludes the possibility of investigating the MR hysteresis through continuous field sweeping at different sweeping rates. Besides, the trapped flux inside the magnet also leads to a hysteresis of ~0.05T. No significant MR hysteresis within this measurement precision was observed.

Compound	Lattice constant (Å)
$Bi_2Ir_2O_7$	10.320
$Y_2Ti_2O_7$	10.090
$Dy_2Ti_2O_7$	10.131

Supplementary Table 1: Lattice constant. Lattice constant of the pyrochlore compounds

Sample Name	Formula	Film Thickness (nm)	Field Value at which the MR Anomaly Peak Occurs (T)	FWHM (T)	MR peak amplitude (%)	Relative peak amplitude (%)
BD1	$Bi_2Ir_2O_7$/ $Dy_2Ti_2O_7$	3.4	1.56	0.359	0.622(5)	0.690(6)
BD2	$Bi_2Ir_2O_7$/ $Dy_2Ti_2O_7$	3.2	1.61	0.276	1.098(6)	1.231(7)
BD3	$Bi_2Ir_2O_7$/ $Dy_2Ti_2O_7$	3.9	2.03	0.540	0.912(3)	0.981(3)
BD4	$Bi_2Ir_2O_7$/ $Dy_2Ti_2O_7$	4.0	1.60	0.601	0.184(5)	0.195(5)
BD5	$Bi_2Ir_2O_7$/ $Dy_2Ti_2O_7$	23.8	1.65	0.220	0.060(5)	0.062(5)
BD6	$Bi_2Ir_2O_7$/ $Dy_2Ti_2O_7$	41.4				
BY1	$Bi_2Ir_2O_7$/ $Y_2Ti_2O_7$	3.7				

*MR peak amplitude = MR at the anomaly peak - background MR
*Relative peak amplitude = (MR at the anomaly peak – background MR) / |background MR|

Supplementary Table 2: Sample Information. List of synthesized heterostructures and details about their observed MR anomaly. The anomaly of each sample was refined with a Gaussian function to extract the position and the full-width-at-half-maximum (FWHM). The absolute and relative MR anomaly amplitude were also calculated and provided in the last two columns. The field direction of the BD3 sample has a 5-degree offset which causes the anomaly to appear at a slightly higher field.

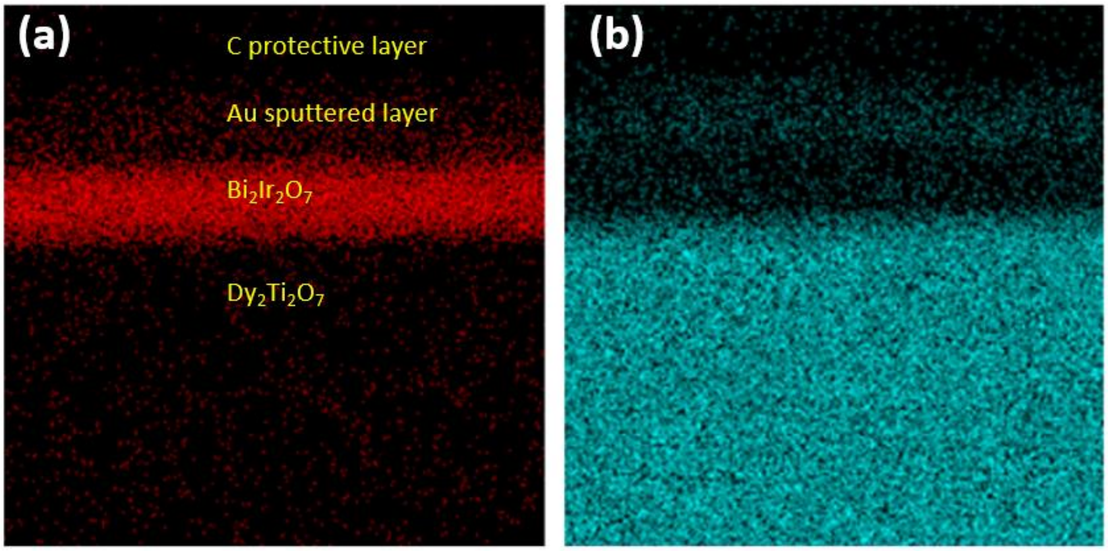

Supplementary Figure 1: Energy dispersive spectroscopy images. (**a**) Bi EDS map; (**b**) Dy EDS map. The EDS results demonstrate that there is no significant interdiffusion across the interface.

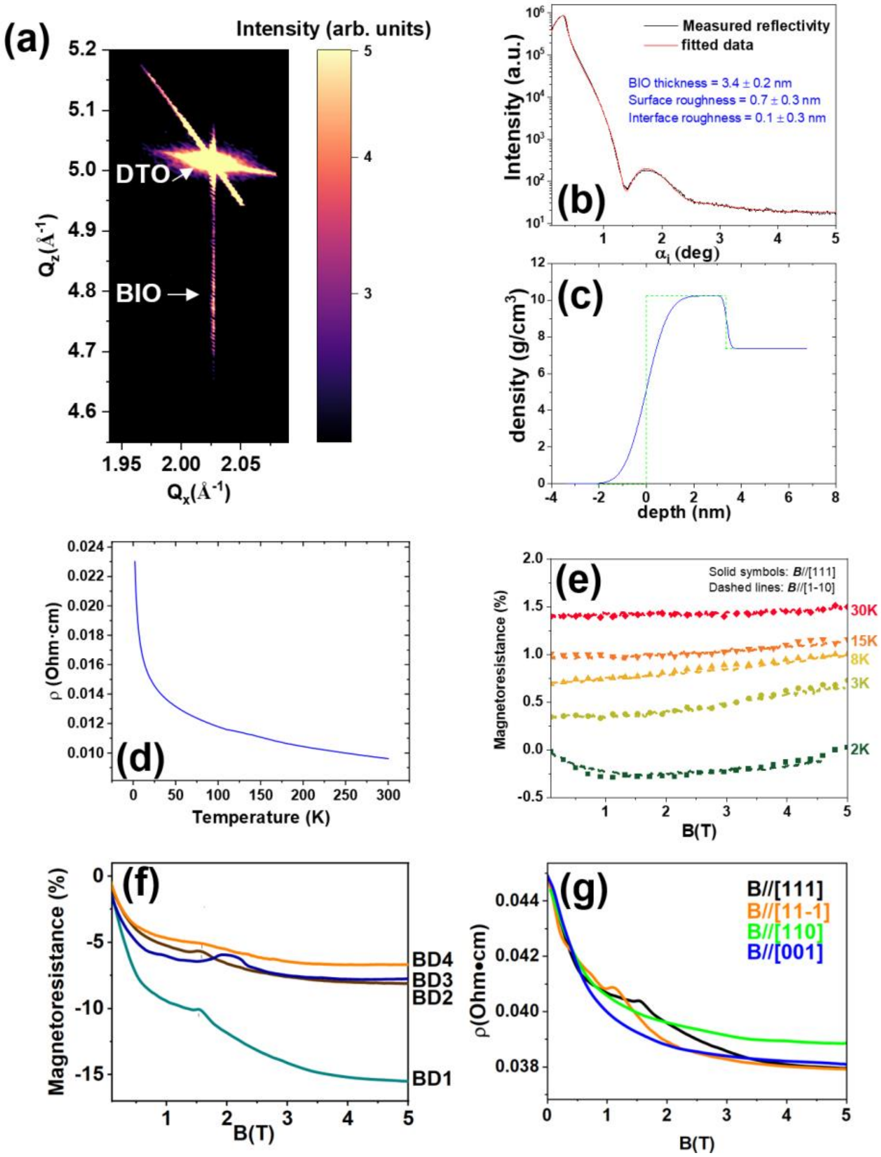

Supplementary Figure 2: Additional characterization of the BIO/DTO (BD1) heterostructure. (a) Reciprocal Space Mapping near the DTO [266] reflection, showing the BIO film is fully strained. Here, Q_x and Q_z are the components of the reciprocal space vector along the [-211] and [111] directions, respectively. (**b**) Representative XRR data. (**c**) The blue curve shows the resulted depth profile of mean density (i.e. laterally averaged). The depth profile for the undisturbed system prior to including roughness is plotted by the dashed green curve indicating the positions of the surface and the layer/substrate interface. (**d**) Temperature-dependent resistance from 300 K down to 2K. (**e**) Magnetoresistance from 30K to 2K is similar between **B**//[111] and **B**//[1-10]. A vertical offset was applied for clarity purpose. (**f**) Magnetoresistance for BIO/DTO (BD1) heterostructures compared with BD2, BD3 and BD4 of similar BIO thickness at 0.03K. The field direction of the BD3 sample has a 5-degree offset which causes the anomaly to appear at a slightly higher field. (**g**) Magneto-transport measurement reproduced from Fig. 2d in the main text without the vertical offset, where the magnetoresistance shown in percentage ratio is replaced by the measured resistivity here.

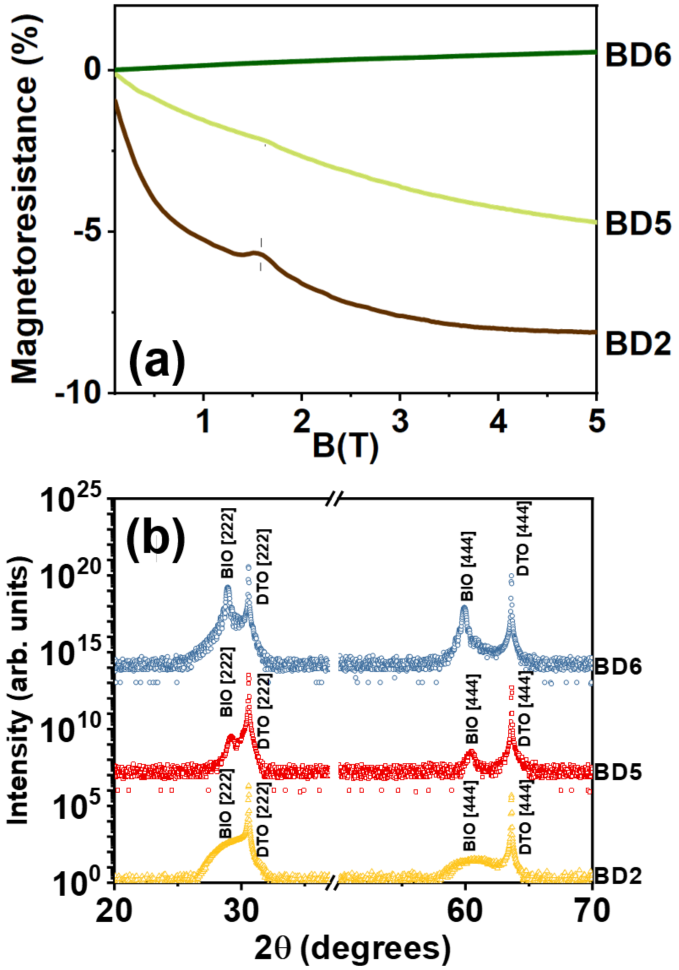

Supplementary Figure 3: BIO/DTO heterostructures with different BIO thickness. (**a**) Magnetoresistance at 0.03K for the BD2, BD5 and BD6 heterostructures, respectively. (**b**) Theta-2theta scans for samples BD2, BD5 and BD6.

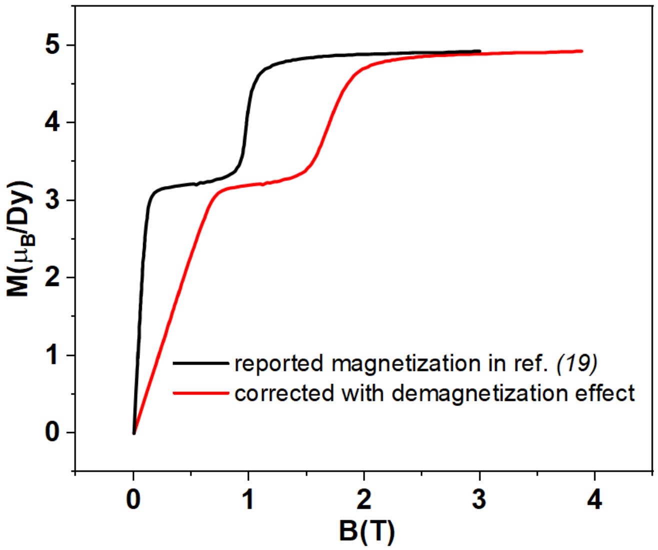

Supplementary Figure 4: Correction of the demagnetization field. The reported magnetization[19] of bulk DTO with **B**//[111] at 0.48 K (black) is used to estimate the demagnetization effect, where the demagnetization factor is calculated to be 0.84 based on the dimensions of the DTO substrate (see Materials and Methods). It can be seen that, when plotting the magnetization against the total external field applied (red), the original ice-rule-breaking transition at 1 T is shifted to ~1.5 T, consistent with the reported MR anomaly in this work.

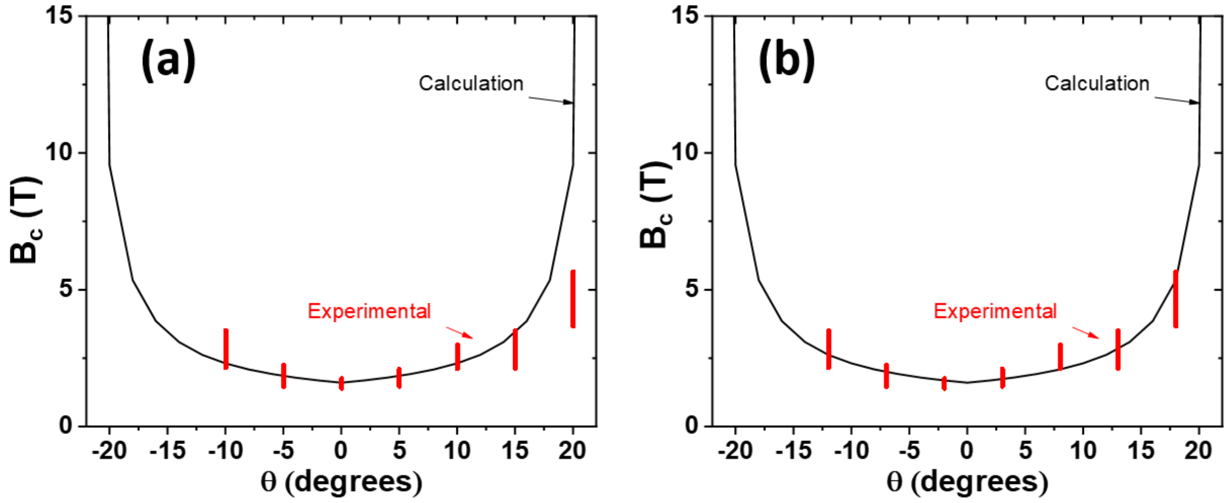

Supplementary Figure 5: Shift of the critical field. (**a**) Shift of the MR anomaly when the magnetic field deviates from the [111] axis (extracted from Figure 2b); and (**b**) the result after correcting a 2° misalignment. The red vertical lines mark the region of the MR anomaly at each field direction.

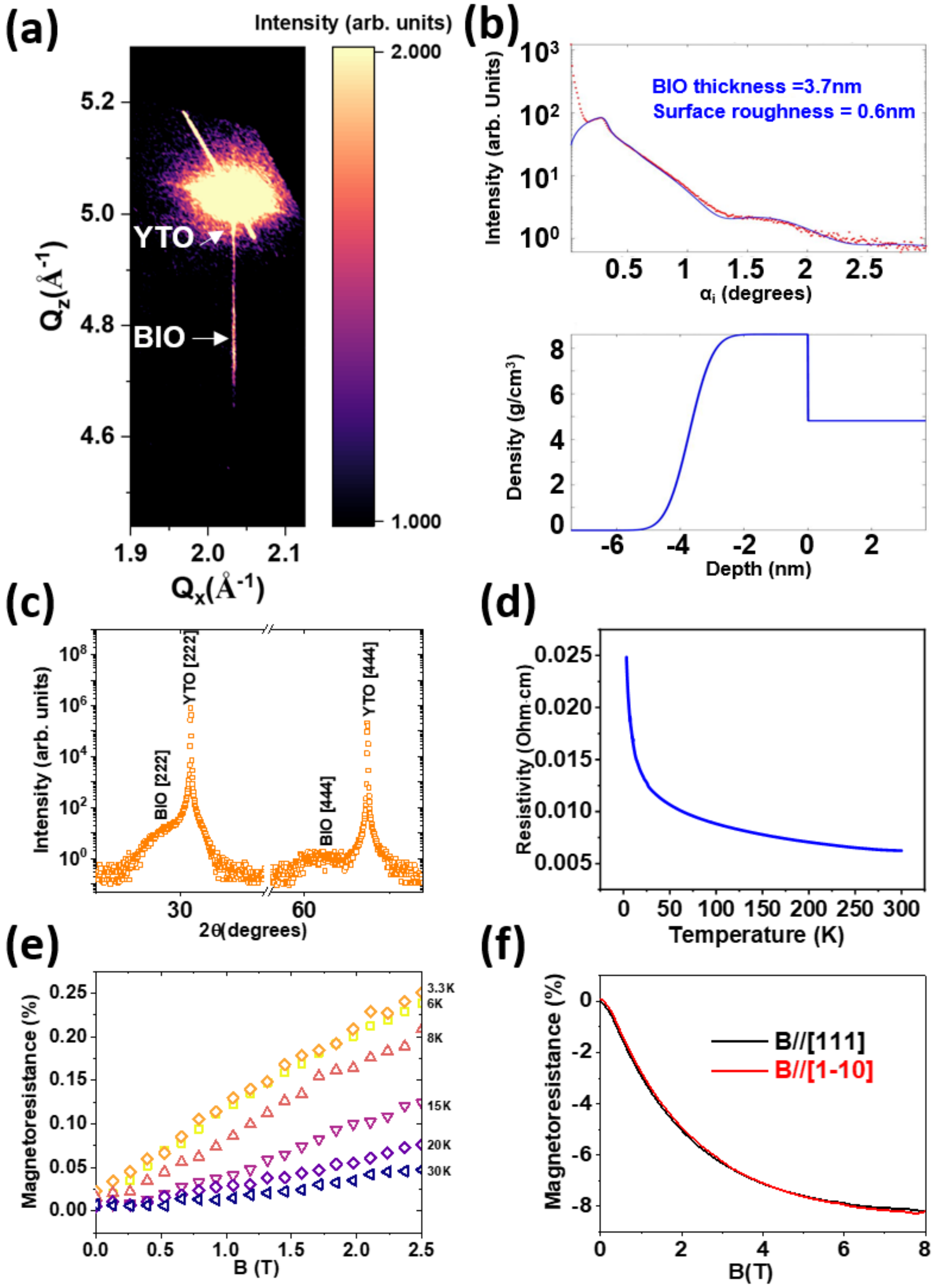

Supplementary Figure 6: Characterization of the $Bi_2Ir_2O_7/Y_2Ti_2O_7$ (BY1) heterostructure. (**a**) Reciprocal Space Mapping near the YTO [266] reflection, showing the BIO film is fully strained. Here, Q_x and Q_z are the components of the reciprocal space vector along the [-211] and [111] directions, respectively. (**b**) Representative XRR data and the resulted depth profile. (**c**) Theta-2theta scans covering the YTO [222] and [444] peaks. (**d**) Temperature-dependent resistivity and (**e**) Magnetoresistance above 2K. (**f**) Magnetoresistance at 0.03K. No difference is observed when the magnetic field **B** is applied along [111] (black) and [1-10] (red).
